# Are Ethiopian schools of medicine producing competent medical graduates for providing quality health care in the era of COVID-19 pandemic?

**DOI:** 10.1186/s12909-023-04510-y

**Published:** 2023-07-19

**Authors:** Dereje Bedane, Gebremariam Getaneh, Gebeyehu Tsega

**Affiliations:** 1 School of Medicine, College of Medicine and Health sciences, Bahir Dar University, Bahir Dar, Ethiopia; 2 Department of Health Systems Management and Health Economics, School of Public Health, College of Medicine and Health sciences, Bahir Dar University, Bahir Dar, Ethiopia

**Keywords:** Competence, Licensure examination, Competency-based education, Ethiopia

## Abstract

**Background:**

Competent health workforce, including medical doctors, is the heart of health systems. Cognizant of this, Ethiopia is implementing licensure exam as a strategy to produce competent health workforce, including medical doctors and beyond, for the provision of high quality health care, among others. However, there is a dearth of evidence on medical graduates’ competence in Ethiopia in the era of Covid-19 pandemic. Hence, this study aimed to assess the competence of medical graduates-based on licensure exam results in Ethiopia.

**Methods:**

A multi –center institution-based cross-sectional study was conducted among 1051 medical graduates (selected through cluster sampling method) from May - July 2022 in Medical Schools found in Amhara region, Northwest Ethiopia. Data were collected from secondary sources at the Ministry of Health and Medical Schools using a structured checklist. Data analysis was performed using SPSS Version 23 software. A binary logistic regression analysis was performed to identify factors associated with graduates’ competence.

**Results:**

Nine hundred sixty-one (91.4%) medical graduates were competent. The study revealed that those graduates with older age (AOR: 0.63; 95% CI: 0.52, 0.76), being female graduates (AOR: 0.39; 95% CI: 0.22, 0.69), graduated in 2021 (AOR: 0.31; 95%; CI: 0.17, 0.60) and attending education in junior medical schools (AOR: 0.06; 95% CI : 0.01, 0.40) have lower competence as compared with that of their counterparts. Whereas, graduates with no repeating internship attachment (AOR: 2.41; 95% CI: 1.40, 4.17) and graduates with repeating academic year (AOR: 2.01; 95% CI: 1.14, 3.56) have better competence than that of their counterparts.

**Conclusion:**

The proportion of competent medical graduates was relatively low as per the national strategic plan which aspires that all medical graduates to be competent. Medical graduate’s competence was affected by age, gender, curriculum being implemented, and having academic as well as internship repeats. As result, policymakers should scale up competency based education in Medical Schools.

## Introduction

Medical graduate’s competence is the state of proficiency of a medical graduate to perform the required health care practice to the defined quality [1]. Competent health workforce, including medical doctors, is the heart of health systems. As a result, producing competent medical graduates(through quality medical education) is a global, regional and national agenda as indicted in Sustainable Development Goals(SDGs), Agenda 2063 of Africa and Ethiopian Health Sector Transformational Plan two(HSTPII)[2–4].

Quality and adaptive medical education is vital for high quality health care provision and positive health outcomes. The quality of health care that the public receives is determined by the quality of medical education [1]. Literature stated that the context, content, and conditions of the social effort to educate competent medical graduates are dynamic and complex [2, 5, 6]. For example, medical education substantially changed during the COVID-19 pandemic [7–10].

The primary goal of medical education is to produce physicians who meet the current and future health needs and expectations of individuals and community [1, 2, 11]. However, medical education has not kept pace to meet these needs because of fragmented, outdated, and static curricula that produce ill equipped medical graduates [6]. There is also a systemic problem such as mismatch of competencies to patients and population needs [12].

Keeping this in mind, different countries, including Ethiopia, promote competency based medical education (CBME) and apply a licensure exam strategy to identify competent medical graduates for the provision of quality and safe health care [13–15].

Ethiopia has 28 public Medical Schools, embedded with universities [16, 17]. These Medical Schools have intended to transform medical educational approach through competency-based medical education (CBME)[18]. Competency-based education is an outcome based and a promising approach to align educational strategies with health needs of the population and health systems demands [1, 19, 20]. Integrating competencies in to a curriculum, putting students at the center and co-production of competencies among facilitators and students are basic principles of CBME [9, 21].

The government of Ethiopia is committed to strengthen the resilience of medical education to produce competent medical graduates. As part of this commitment, a “New Innovative Medical Education Initiative (NIMEI)” and CBME-based on the Canadian Medical Education Directives for Specialists (Can-MEdS) competency framework- were introduced in Ethiopia since 2012 and 2015, respectively [14, 22, 23]. The Can-MEdS framework categorized competencies into 7 major domains: medical expert, professional, leader, communicator, collaborator, health advocator, and scholar [24].

Despite this effort, changes such as the triple threats-COVID-19, conflict, and climate change- imposed additional challenges on Ethiopian medical education, especially at undergraduate level. For example, face to face education was interrupted due to the COVID-19 pandemic [9, 25, 26]. As such, the competence of medical graduates is not satisfactory to meet the existing and future health needs of the people [27, 28]. This leads to weak and fragile health system to respond to health emergencies, along with the existing health needs of the population and health systems demands. This is reflected during the COVID-19 pandemic [29].

Moreover, Ethiopia’s health outcomes, as measured with the standard indictors, are remaining poor as compared with other African countries [3, 15]. This is, partly, due to incompetent medical doctors, as a result of poor quality medical education. Incompetent medical graduates provide low quality health care that harm patients and population, including disability and death. Such low quality health care also leads to high economic burden for the patients and the country [30, 31].

Flooding policy (regardless of their competence) and lack of regular curriculum revision in medical education, among others, are the common causes of incompetent medical graduates [32, 33]. As such, a reputability of medical schools in general, medical professions in particular will be lost [34, 35]. However, there is a dearth of evidence on medical graduates’ competence in Ethiopia in the era of Covid-19 pandemic. Hence, this study aimed to assess the competence of medical graduates-based on licensure exam results in Ethiopia.

## Methods

### Study area and period

The study was conducted from May 2022 to July 2022, in four randomly selected public universities in the Amhara region: Bahir Dar university, university of Gondar, Debre Tabor university, and Debre Markos university.

### Study design

A multi-center institution based cross-sectional design was conducted among medical student graduates who took the national licensure exam in medical schools found in the Amhara region, Northwest Ethiopia.

### Source population

The source population was all medical student graduates who took the national licensure exam in medical schools found in the Amhara region, Northwest Ethiopia during the study period.

### Study population

All medical student graduates who took the national licensure exam in randomly selected medical schools were found in the Amhara region, Northwest Ethiopia during the study period.

### Inclusion and exclusion criteria

#### Inclusion criteria

All medical graduates who took the national licensure exam for the first time were included in the study.

#### Exclusion criteria

Medical graduates who took the national licensure exam two or more times were excluded from the study.

### Study variables

#### Dependent variable

- Medical graduates’ competence.

#### Independent variable

Socio-demographic factors.

- Age.
- Sex.

Student profile at graduation.

- CGPA
- Having academic year repeat
- Having internship repeat
- Year of graduation

University-related factors

- Classroom size
- Student size for clinical attachments
- Seniority of Medical school
- Type of curriculum being implemented
- Book to student ratio

### Operational definition

#### Competence

medical graduate’s competence measured by his/her result in the national licensure exam. If the graduate passed the exam, s/he considered as competent otherwise incompetent.

#### Academic year repeat

is when the graduate has at least a year repeat or course repeat during his/her first 5-year stay.

#### Internship repeat

is when the graduate has at least an attachment repeat from the four major attachments during his/her internship period.

#### Class size

is the number of medical students per class during the lecture as compared to the standard red class size (40 students per class) per the Ministry of education guideline.

#### Student size for clinical attachments

is the number of students per group for clinical attachments either bedside or round as per each medical school implementation.

#### Seniority of medical school

classification (1st generation, 2nd generation, 3rd generation) of Medical Schools by the Ministry of Education based on year of establishment.

#### Type of curriculum being implemented

is the type of curriculum being implemented in the medical school. It can be competency-based, New Innovative Medical Education Initiative (NIMEI), or conventional medical curriculum.

### Sample size determination

The sample size was determined based on the single population proportion formula by using the following assumptions:

$$\text{n}=\frac{(\text{Z}\text{a}/2)2 \text{P}\left(1-\text{P}\right)}{\text{d}2}$$

Z = 95% confidence interval (1.96).

P = 50% (proportion of medical graduates who pass the exam taken as 0.05 since there is no literature).

d = 0.03 (margin error).

$$n = \frac{{{{(1.96)}^2}*0.5(1 - 0.5)}}{{{{(0.03)}^2}}} = 1067$$

### Sampling technique and procedure

The cluster sampling technique was employed to select the medical school and the study participants. After medical schools were randomly selected all medical graduates who took the licensure exam for the first time were included in the study.

### Data collection procedure and tools

Data were retrieved from secondary sources using a structured checklist for data extraction that was created based on various related literature. Graduates’ licensure exam results, age, and sex were retrieved from Health Professionals’ Competency Assessment & Licensure Directorate of Ethiopian Ministry of Health. The other variables were retrieved from the Medical Schools.

### Data quality assurance

To ensure quality of data, the questionnaire was pretested on 5% of the sample size. The data collectors and supervisors were trained for 3 days. Strict supervision during field work was conducted. Data completeness was checked and data cleaning was done after field work.

### Data processing and analysis

After cleaning and checking data completeness, data were entered into SPSS software version 23.0. Graduate’s competence was measured by whether they scored the pass mark on the licensure exam. Both descriptive and inferential statistics were used. The descriptive statistics was used to describe the frequency, central tendency and dispersion of the data. Binary logistic regression was used to identify the predictors of medical graduates’ competence. All assumptions of binary logistic regression were checked, some transformation was done accordingly. Model fitness was checked by Hosmer and Lemeshow test. The odds ratio (OR) was used to measure the strength of association. Predictor variables with P < 0.05 were considered statistically significant factors for graduates’ competence.

## Results

### Socio-demographic characteristic medical graduates

The response rate was 98.5%. From the medical graduates, 196 (18.6%), 619(58.9% and 236(22.5%) took the exam in the year 2019, 2020 and 202, respectively. More than three fourth (75.5%) study participants were male and the mean age of the study participants is 26.5 (± 3 SD) with a minimum age of 23 and maximum age of 38. Of the participants, 58 (5.52%) graduates scored a CGPA of less than or equal to 2.49, 472 (44.91%) graduates scored from 2.50 to 2.99 CGPA, 463 (44.05%) graduates scored from 3.00 to 3.49 and 58 (5.52%) graduates scored greater than or equal to 3.5 with an overall mean of 2.99 (± 0.3SD). Regarding year of graduation, 630 (59.9%) and 421(40.1%) of the study participants graduated in 2020/21 and in 2019/20, respectively. From the study participants, 161 (15.32%) of them had repeated attachment during his/her internship and 260 (24.74%) of the study participants had at least one year academic repeat or course repeat during their stay on campus (Table 1).

**Table 1:** Sociodemographic characteristics of medical graduates, Ethiopia, 2022

Variable	Categories	Frequency	Percentage
Sex	Female	258	24.55%
	Male	793	75.45%
CGPA	Less than or equal to 2.49	58	5.52%
	From 2.5 to 2.99	472	44.91%
	From 3.0 to 3.49	463	44.05%
	Greater than or equal to 3.5	58	5.52%
Graduation year	2020/21	630	59.94%
	2019/20	421	40.06%
More than one internship attachment	No	890	84.68%
	Yes	161	15.32%
Academic year repeating	No	791	75.26%
	Yes	260	24.74%

### Profiles of medical graduates in medical schools

Among 1051 medical graduates, 112(10.7%), 80 (7.6%), and 859 (81.7%) have completed their education with NIMEI, competency-based and conventional medical curricula, respectively. More than half of medical graduates, 572(54.4%), graduated in first-generation medical schools. Most of the graduates, 971 (92.4%) educated with higher class size than the standard class size of medical students which is 40(Table 2).

**Table 2:** Medical Schools’ characteristics, Ethiopia, 2022

Variable	Categories	Frequency	Percentage
Curriculum being implemented	NIMEI	112	10.7%
	Competency-Based	80	7.6%
	Conventional	859	81.7%
Medical school seniority	First generation	192	18.3%
	Second generation	287	27.3%
	Third generation	572	54.4%
Average number of students per class	Less or equal to 40	80	7.6%
	Greater than 40	971	92.4%
Average number of students per group for clinical attachment	6 Students	80	7.6%
	12 Students	399	38.0%
	15 Students	572	54.4%

### Competence of medical graduates

Nine hundred sixty-one (91.4%) medical graduates were competent. Two hundred thirty three (90.3%) of female and 728 (91.8%) of male medical graduates were competent. From medical graduates of 2019/20, 405 (96.2%) of them were competent. Almost all, 79 (98.8%, medical graduates with competency based curriculum were competent (Table 3).

**Table 3:** Competence of medical graduates in Medical Schools in Ethiopia, 2022

Variables		Competent	
		Yes	No
		Number (%)	Number (%)
Sex	Female	233 (90.3%)	25 (9.7%)
	Male	728 (91.8%)	65 (8.2%)
Academic year repeating	No	751 (94.9%)	40 (5.1%)
	Yes	210 (80.8%)	50 (19.2%)
More than one internship attachment	No	828 (93.0%)	62 (7.0%)
	Yes	133 (82.6%)	28 (17.4%)
Graduation year	2020/21	556 (88.3%)	74 (11.7%)
	2019/20	405 (96.2%)	16 (3.8%)
CGPA	Less than or equal to 2.49	39 (67.2%)	19 (32.8%)
	From 2.5 to 2.99	406 (86.0%)	66 (14.0%)
	From 3.0 to 3.49	458 (98.9%)	5 (1.1%)
	Greater than or equal to 3.5	58 (100.0%)	0 (0.0%)
Curriculum being implemented	NIMEI	91 (81.3%)	21 (18.8%)
	Competency based	79 (98.8%)	1 (1.3%)
	Conventional	791 (92.1%)	68 (7.9%)

### Factors associated with medical graduates’ competence

From the multiple logistic regression age, sex, year of graduation, having more than one internship attachment, having academic year repeat and medical school seniority were statistically significant factors in graduates’ competence.

The study stated that medical graduate’s likelihood of being competent decreases by 37% (AOR: 0.63; 95% CI: 0.52, 0.76) as their age increases by one year. Additionally, compared to male graduates, female graduates were 61% (AOR: 0.39; 95% CI: 0.22, 0.69) less likely to be competent. Regarding to year of graduation, compared to medical graduates of 2019/20, medical graduates of 2020/21 were 69% (AOR: 0.31; 95%; CI: 0.17, 0.60) less likely to be competent. Compared to medical graduates of first-generation medical school, medical graduates from third and second generation medical schools were 94% (AOR: 0.06; 95% CI: 0.01, 0.40) less likely to be competent (Table 4).

The study also found that medical graduates who had no repeats were 2.41 (AOR: 2.41; 95% CI: 1.40, 4.17) times more likely to be competent as compared with that of medical graduates who had at least one internship repeat. Moreover, compared to medical graduates who had an academic year repeat, those who did not have the repeat were 2.01 (AOR: 2.01; 95% CI: 1.14, 3.56) times more likely being competent (Table 4).

**Table 4:** Factors associated with medical graduates’ competence, Ethiopia, 2022

		Competent				
		Yes	No			
Variables	Categories	Number (%)	Number (%)	COR (95% CI)	AOR (95% CI)	P- value
Age(in years)				0.83 (0.79, 0.88)	0.63 (0.52, 0.76)	< 0.001
Sex	Female	233 (24.2%)	25 (27.8%)	0.83(0.51, 1.35)	0.39 (0.22, 0.69)	0.001
	Male	728 (75.8%)	65 (72.2%)	1	1	
Year of graduation	2020/21	556 (57.9%)	74 (82.2%)	0.30 (0.17, 0.52)	0.31 (0.17, 0.60)	< 0.001
	2019/20	405 (42.1%)	16 (17.8%)	1	1	
More than one internship attachment	No	828 (86.2%)	62 (68.9%)	2.81 (1.74, 4.55)	2.41 (1.40, 4.17)	0.002
	Yes	133 (13.8%)	28 (31.1%)	1	1	
Academic year repeating	No	751 (78.1%)	40 (44.4%)	4.47 (2.87, 6.96)	2.01 (1.14, 3.56)	0.016
	Yes	210 (21.9%)	50 (55.6%)	1	1	
Medical school seniority	First generation	170 (17.7%)	22 (24.4%)	1	1	
	s generation	263 (27.4%)	24 (26.7%)	1.42 (0.77, 2.61)	0.06 (0.01, 0.38)	0.003
	Third generation	528 (54.9%)	44 (48.9%)	1.55 (0.91, 2.67)	0.06 (0.01, 0.40)	0.003

## Discussion

The study aimed to assess the level of competence and its associated factors among medical graduates in medical schools found in the Amhara region, Northwest Ethiopia. Nine hundred sixty-one (91.4%) medical graduates were competent. When compared to studies done in the United States, Japan, and South Africa, the finding of this study was high, even though the exam’s complexity was different [36–38]. By taking into account the total cost of preparing medical students to be professional physicians, the Ethiopian national startegic plan of health professionals’ competence aspires that all medical graduates to be competent. Our current finding is not bad, though, when the nation considers the “flooding” policy that caused serious educational issues that were not fully resolved by straightforward initiatives. To maintain high standards in medical education and achieve better competence what we currently have, concurrent increases in funding are required for faculty expansion as well as infrastructure development [33].

In the current study, as medical graduates get older, they are less likely to be competent, and female medical students are less likely to be competent which is consistent with that of a previous study conducted in the United States [39]. A long medical school stays may expose to lifestyle diseases like depression, stress and psychological ill health as medical graduates get older. Having a long and demanding medical education can have a negative impact on graduates’ psychological health as they get older, which may explain why older medical students have a lower competence [40–42].

On the other hand, female students in medical school are negatively impacted by gender bias both directly and indirectly. It may hinder their progress, keeping them at a lower grade scale, and it may also have psychological effects that cause low self-esteem and occasionally poor competence. Findings from previous studies stated that 50 to 75% of medical students encountered gender discrimination. The offensive conduct including spreading potential harm, using sexist epithets, and even making sexual advances may affect females. Both faculty and residents are accused of harassing students. Although harassment occurs frequently during medical school, females are more frequently affected by it due to their gender. This issue is thought to be exacerbated by the hierarchical power structure of medicine, which places men in its highest positions [43, 44]. Additionally, female medical students may still struggle with the strain of their dual roles as women and doctors, conflicts between their career and lifestyle choices, and challenges with timing pregnancies that have significant social value [45]. Our findings and those of previous studies indicated that Ethiopia could lessen the impact of gender and older age on academic performance by incorporating age criteria as entry behavior or by providing older students with additional supportive education and more necessities for female students in order to make up for their social and gender-based pressures.

From this study, almost all (98.75%) graduates with the competency-based curriculum were competent. 81% of graduates with NIMEI and 92.08% of graduates with the conventional curriculum were competent which is consistent with that of previous studies conducted in USA [46] and in India [47] Medical Schools.

In Ethiopia, a program called the New Innovative Medical Education Initiative (NIMEI) was started in February 2012 to train medical professionals using a new methodology and curriculum [22, 23]. After the curriculum has been put into practice, the task of instructing medical students is taken in second-generation universities [48] without an adequate number of senior staff members and in the absence of their hospitals. On the other hand, advanced skill labs and senior staff are required for medical education to continue. The success, achievement, and satisfaction of students are significantly influenced by their learning environment including human power [49, 50]. The difficulties that universities face in continuing the program in newly established medical schools may be the cause for why students who followed this program had relatively the lower competence. The study also highlighted the need for strong physical infrastructure, establishing national and international partnerships, and addressing faculty shortages in basic and clinical sciences for the benefit of faculty development at universities that have the program.

In addition, despite the lack of scientific support, the author believes that some things can be done to improve the way that students enter the NIMEI program. Students from other natural sciences, such as mathematics, biology, and chemistry, have joined the program and learned medicine for only five years that affect the quality of medical education [23].

Graduation year is another predictor variable that had a statistically significant association. This may be explained the fact that the COVID-19 pandemic and the country’s recent war affected the quality of medical education. Stress, fear, and anxiety are unavoidable effects of an outbreak of a poorly understood contagious disease (COVID-19) [10]. Due to the pandemic’s ongoing spread, sensational media coverage, and implementation of social lockdown, university students experience negative mental health outcomes [9]. Due to the cancellation and postponement of anticipated events like exchange studies and graduation ceremonies, graduating class students were more negatively impacted [51]. This finding teaches us that students who experience unequivocal environmental and other similar siege require significant psychological and other social treatments.

In the current study, graduates’ competence was also affected by medical schools’ seniority. Medical graduates who graduated from first and second-generation medical schools are 94% more likely to be competent as compared with that of graduates from third generation medical school.

Academic year repetition (during their studies) is yet another element that significantly influenced graduates’ competence. Compared to graduates who had an academic year repeat, those who didn’t have academic year repeat more likely to be competent which is consistent with that of a study done in Kenya [52].

In addition to repeating an academic year, having more than one internship attachment resulted in significantly lower competent. Academic-related stressors are the main cause of poor competence such as repeats for medical students in medical school. These stressors include not having enough time to review what has been learned, having disagreements with teachers, not knowing what is expected of them, having to deal with patients who are ill or dying, not wanting to study medicine, and feeling pressured to perform poorly. Additionally, those with repeated internship attachment may had to deal with more stress because they felt guilty for wasting more time and settling a long medical journey. They might perform worse academically as a result [53].

Secondly, with a few exceptions, such as students who repeat due to poor conduct or students who fail to give informal things like sexual benefits to teachers, the majority of students who repeat during an internship or in prior year studies are those who have low academic performance. Therefore, compared to students who have not yet repeated classes, it is indeed a reality for students with a history of failing classes will perform academically worse on licensure exams. The present report suggested that medical academic institutions are in charge of supporting education for those students experiencing academic repeat and looking into the causes of academic repeat during internship and prior year studies.

## Conclusion

The competence of medical graduates was relatively low per the national strategic plan which aspires that all medical graduates to be competent. Medical graduate’s competence was affected by age, gender, curriculum being implemented, and having academic as well as internship repeats.

### Recommendations

#### For medical schools

- Medical Schools should give especial emphasis for older and female medical students and those students who have more than one internship attachment to improve medical graduates’ competence.
- Medical Schools should scale up CBME.

#### For policymakers

- Policymakers should consider strategy to scale the competency-based curriculum in all Medical Schools.

#### For researchers

- Longitudinal study with more potential variables that may affect medical graduates’ competence should be conducted.

## Data Availability

Please contact corresponding author for data requests.

## List of abbreviations

CBME: Competency-Based Medical Education
CGPA: Cumulative Grade Point Average
MoH: Ministry of Health
NIMEI: New Innovative Medical Education Initiative
SPSS: Statistical Package for Social Science
USA: United States of America
WHO: World Health Organization

## References

[1] World Health Organization. Global competency and outcomes framework for universal health coverage. World Health Organization; 2022. Mar 31.

[2] Frenk J, Chen L, Bhutta ZA, Cohen J, Crisp N, Evans T, Fineberg H, Garcia P, Ke Y, Kelley P, Kistnasamy B. Health professionals for a new century: transforming education to strengthen health systems in an interdependent world. The lancet. 2010 Dec 4;376(9756):1923-58. 10.1016/S0140-6736(10)61854-5 21112623

[3] FMOH. Health Sector Transformation Plan two(HSTPII), Addis Ababa, Ethiopia. 2021.

[4] DeGhetto K, Gray JR, Kiggundu MN. The African Union’s Agenda 2063: Aspirations, challenges, and opportunities for management research. Afr J Manage 2016 Jan 2;2(1):93–116.

[5] Sallis E. Total quality management in education. Routledge; 2014.

[6] Skochelak SE, Lomis KD, Andrews JS, Hammoud MM, Mejicano GC, Byerley J. Realizing the vision of the Lancet Commission on Education of Health Professionals for the 21st Century: Transforming medical education through the Accelerating Change in Medical Education Consortium. Medical Teacher. 2021 Apr 8;43(sup2):S1-6. 10.1080/0142159X.2021.1935833 34291718

[7] Fund C. Training tomorrow’s doctors: The medical education mission of academic health centers. Commonwealth Fund; 2002.

[8] Karle H, Gordon D (2007). Quality standards in medical education. The Lancet.

[9] Frenk J, Chen LC, Chandran L, Groff EO, King R, Meleis A, Fineberg HV. Challenges and opportunities for educating health professionals after the COVID-19 pandemic. The Lancet. 2022 Oct;29(10362):1539–56. 10.1016/S0140-6736(22)02092-X PMC9612849 36522209

[10] Dedeilia A, Papapanou M, Papadopoulos AN, Karela NR, Androutsou A, Mitsopoulou D, Nikolakea M, Konstantinidis C, Papageorgakopoulou M, Sideris M, Johnson EO. Health worker education during the COVID-19 pandemic: global disruption, responses and lessons for the future—a systematic review and meta-analysis. Hum Resour Health. 2023 Dec;21(1):1–35. 10.1186/s12960-023-00799-4 PMC9951171 36829158

[11] Sheriff DS (2020). Competency-based Medical Education in India. Annals of SBV.

[12] Organization WH. Education and training. 2016.

[13] Hertz NR, Chinn RN. Licensure examinations. Council on licensure, enforcement and regulations [CLEAR]. 2000.

[14] Ethiopia FMOH. Strategic Plan For Health Professionals’ Competency Assessment and Licensing, 2021–2025, Addis Ababa, Ethiopia. 2021.

[15] FMOH., a Roadmap for Continuing Professional Development for Health Professionals in Ethiopia,2020–2026, Addis Ababa, Ethiopia. 2020.

[16] Career ESPfE. List of Medical Colleges in Ethiopia 2021: Ethiopia National Educational Assessment and Examinations Agency. ; 2021 [Available from: https://www.neaea.com/list-of-medical-colleges-in-ethiopia/.

[17] FMOH, National Specialty and Sub-Specialty Service Roadmap,2020–2029, Addis Ababa, Ethiopia. 2020.

[18] Scheopner Torres A, Brett J, Cox J. Competency-based learning: definitions, policies, and implementation. Education Development Center Inc; 2015.

[19] Burnette DM (2016). The renewal of competency-based education: a review of the literature. J Continuing High Educ.

[20] Nodine TR (2016). How did we get here? A brief history of competency-based higher education in the United States. J Competency‐Based Educ.

[21] McGaghie WC, Sajid AW, Miller GE, Telder TV, Lipson L, Organization WH. Competency-based curriculum development in medical education: an introduction. World Health Organization; 1978. 664734

[22] Abraham Y, Azaje A (2013). The new innovative medical education system in Ethiopia: background and development. Ethiop J health Dev.

[23] Gebru HT, Verstegen D. Assessing predictors of students’ academic performance in ethiopian new medical schools: a concurrent mixed-method study. BMC Med Educ. 2023 Dec;23(1):1–9. 10.1186/s12909-023-04372-4 PMC10276416 37330493

[24] Education FDRoE-MoHiCwMoSaH. Competency- Based, Integrated, Modular Medical Curriculum Revised 2021.

[25] Densen P (2011). Challenges and opportunities facing medical education. Trans Am Clin Climatol Assoc.

[26] Mennin S (2021). Ten global challenges in medical education: wicked issues and options for action. Med Sci Educ.

[27] Faustinella F, Jacobs RJ (2018). The decline of clinical skills: a challenge for medical schools. Int J Med Educ.

[28] Buja LM (2019). Medical education today: all that glitters is not gold. BMC Med Educ.

[29] Gukas ID (2007). Global paradigm shift in medical education: issues of concern for Africa. Med Teach.

[30] Organization WH. Health workforce requirements for universal health coverage and the sustainable development goals.(human resources for health observer, 17). 2016.

[31] Morgan C, Teshome M, Crocker-Buque T, Bhudia R, Singh K (2018). Medical education in difficult circumstances: analysis of the experience of clinical medical students following the new innovative medical curriculum in Aksum, rural Ethiopia. BMC Med Educ.

[32] Tamrat W. Medical Education and the Ethiopian Exodus of Talent. Higher Education in Ethiopia: Brill; 2022. p. 134-6.

[33] Kelly CM, Vins H, Spicer JO, Mengistu BS, Wilson DR, Derbew M (2019). The rapid scale up of medical education in Ethiopia: medical student experiences and the role of e-learning at Addis Ababa University. PLoS ONE.

[34] Nasef A, Al-Griw MA, El Taguri A (2020). Improving quality of education in extreme adversities-the case of Libya. J Biology Med.

[35] Kigonya E (2004). Medical education in Uganda-A critique. East and Central African Journal of Surgery.

[36] EDUCATIONS WTG. UNITED STATES MEDICAL LICENSING EXAMINATION (USMLE). [Available from: https://www.globaleducations.net/united-states-medical-licensing-examination.html#about-us.

[37] Tsunekawa K, Suzuki Y, Shioiri T (2020). Identifying and supporting students at risk of failing the National Medical Licensure examination in Japan using a predictive pass rate. BMC Med Educ.

[38] Kwazulu-Natal uo. Foreign Doctors Sit for SA Exam at Medical School 2016 [Available from: https://ukzn.ac.za/news/foreign-doctors-sit-for-sa-exam-at-medical-school/.

[39] Gauer JL, Jackson JB (2018). Relationships of demographic variables to USMLE physician licensing exam scores: a statistical analysis on five years of medical student data. Adv Med Educ Pract.

[40] Souza IMDM, Paro HBMdS, Morales RR (2012). Pinto RdMC, Silva CHMd. Health-related quality of life and depressive symptoms in undergraduate nursing students. Rev Latinoam Enferm.

[41] Mahawar P, Phadnis S, Ghosh G, Kataria O, Dixit S. Psychological morbidity in students of medical college and science and art college students-A comparative study. Online J health allied Sci. 2011;10(2).

[42] Das P, Basu M, Dasgupta U, Roy B, Das PK, Mundle M (2013). Health related quality of life among undergraduate medical students of Kolkata. Healthline.

[43] Binder R, Garcia P, Johnson B, Fuentes-Afflick E (2018). Sexual harassment in medical schools: the challenge of covert retaliation as a barrier to reporting. Acad Med.

[44] StateUniversity.com. Opportunities for Women- Unique Challenges For Women In Medicine [Available from: https://careers.stateuniversity.com/pages/100000610/Opportunities-Women-UNIQUE-CHALLENGES-WOMEN-IN-MEDICINE.html#ixzz7adZY75S8.

[45] Mobilos S, Chan M, Brown JB (2008). Women in medicine: the challenge of finding balance. Can Fam Physician.

[46] Blake RL, Hosokawa MC, Riley SL (2000). Student performances on step 1 and step 2 of the United States Medical Licensing Examination following implementation of a problem-based learning curriculum. Acad Med.

[47] Pandit S, Thomas MR, Banerjee A, Angadi M, Kumar S, Tandon A (2019). A crossover comparative study to assess efficacy of competency based medical education (CBME) and the traditional structured (TS) method in selected competencies of living anatomy of first year MBBS curriculum: a pilot study. Med J armed forces india.

[48] Research GFfMEa. Medical schools in Ethiopia. [Available from: https://www.gfmer.ch/Medical_search/Countries/Ethiopia.htm.

[49] Badsar A, Taramsari MR, Hoseinpour J, Jahromi SK (2012). Postgraduate Trainees’ perception of the clinical learning environment at an Iranian Medical Sciences University. Procedia-Social and Behavioral Sciences.

[50] Assefa T, Haile Mariam D, Mekonnen W, Derbew M (2017). Medical students’ career choices, preference for placement, and attitudes towards the role of medical instruction in Ethiopia. BMC Med Educ.

[51] Mekonen EG, Workneh BS, Ali MS, Muluneh NY (2021). The psychological impact of COVID-19 pandemic on graduating class students at the University of Gondar, Northwest Ethiopia. Psychol Res Behav Manage.

[52] Nyangena E, Getanda A, Ngugi S. Factors influencing success of bachelor of science in nursing graduates in nursing council of Kenya licensure examinations. 2013.

[53] Melaku L, Bulcha G. Evaluation and Comparison of Medical Students Stressors and Coping Strategies among Undergraduate Preclinical and Clinical Year Students Enrolled in Medical School of Arsi University, Southeast Ethiopia. Education Research International. 2021;2021.

